# Malaria-Infected Red Blood Cell Analysis through Optical and Biochemical Parameters Using the Transport of Intensity Equation and the Microscope’s Optical Properties

**DOI:** 10.3390/s19143045

**Published:** 2019-07-10

**Authors:** Marcel Akpa Agnero, Kouakou Konan, Zan Guy Christian Stephane Tokou, Yao Taky Alvarez Kossonou, Bienvenue Sylvère Dion, Kenneth Amiga Kaduki, Jérémie Thouakesséh Zoueu

**Affiliations:** 1 Laboratoire de Physique de la Matière Condensée et Technologie, UFR SSMT, Université Félix Houphouët-Boigny, 22 BP 582 Abidjan 22, Côte d’Ivoire; 2 Laboratoire d’Instrumentation d’Image et Spectroscopie, Institut National Polytechnique Félix Houphouët-Boigny (INPH-B), BP 1093 Yamoussoukro, Côte d’Ivoire; 3 Département de Maths-Physique-Chimie, Université Jean Lorougnon Guédé, BP 150 Daloa, Côte d’Ivoire; 4 Department of Physics, University of Nairobi, 30197-00100 Nairobi, Kenya

**Keywords:** malaria diagnosis, refractive index, hemoglobin, topography, transport of intensity equation, point spread function

## Abstract

The accuracy, reliability, speed and cost of the methods used for malaria diagnosis are key to the diseases’ treatment and eventual eradication. However, improvement in any one of these requirements can lead to deterioration of the rest due to their interdependence. We propose an optical method that provides fast detection of malaria-infected red blood cells (RBCs) at a lower cost. The method is based on the combination of deconvolution, topography and three-dimensional (3D) refractive index reconstruction of the malaria-infected RBCs by use of the transport of intensity equation. Using our method, healthy RBCs were identified by their biconcave shape, quasi-uniform spatial distribution of their refractive indices and quasi-uniform concentration of hemoglobin. The values of these optical and biochemical parameters were found to be in agreement with the values reported in the literature. Results for the malaria-infected RBCs were significantly different from those of the healthy RBCs. The topography of the cells and their optical and biochemical parameters enabled identification of their stages of infection. This work introduces a significant method of analyzing malaria-infected RBCs at a lower cost and without the use of fluorescent labels for the parasites.

## 1. Introduction

Malaria is an infectious disease caused by protozoan parasites of the genus *Plasmodium* and is transmitted by the female Anopheles mosquito [1,2]. Infection results in a deterioration of the red blood cells (RBCs). Malaria remains one of the most fatal infectious diseases in the world, particularly in tropical Africa. The accuracy, reliability, speed and cost of the methods used for malaria diagnosis are very important in its treatment and eradication. Clinical diagnosis—where the medical practitioner makes a diagnosis based on patient symptoms—is the least expensive and most widely practiced method. Since these symptoms are not specific to malaria, clinical diagnosis can be erroneous. This has led to an emergence of the parasites’s resistance against anti-malarial drugs such as chloroquin and sulfadoxine-pyrimethamine [3]. Over the years, methods of diagnosis which rely on detection of the parasites in blood have emerged with diagnosis by examination of thick and thin blood smears under an optical microscope considered the gold standard. With this set-up, a microscopist is able to confirm the presence of parasites, quantify them and identify the species. However, this method requires use of fluorescent labels for the parasites. This affects the physiological environment of the cells due to the phototoxicity induced by fluorescent molecules [4]. Furthermore, fluorescent labels provide neither the mass nor the concentration of the target cell. They only provide information indicating the location of the target cell [4]. Moreover, the process is time consuming because of the steps required for fluorescent labeling and interpretation of the information on the slides. Results tend to be subjective as they are dependent on the expertise of the microscopist [5]. Due to these shortcomings, immunochromatographic tests known as fast, have been developed. These tests, which detect the enzymes specific to the parasites, are easy to use and interpret but suffer from issues such as false positive and negative results (problem of sensitivity) [6]. Diagnosis through polymerase chain reaction (PCR) is as an alternative technique that has high sensitivity. However, PCR is only available in specialized laboratories due to its material requirements and very high cost [6]. These shortcomings have triggered research into alternative non-invasive methods of diagnosis, with the primary aim of achieving high sensitivity at low cost. Vibrational spectroscopy (Raman and infrared) is an alternative optical approach to microscopy. However, the technique for this method is dominated by hemozoin detection and diagnosis is hindered by the fact that the earliest stages of infected RBCs contain very little hemozoin. Optical methods based on the sectioning of the RBC into a set of images have long been attempted for malaria detection. This makes it possible to visualize the RBC’s interior details [7,8,9]. In this approach, the refractive index of the cell is one of the intrinsic optical parameters exploited [10,11]. This important parameter excludes the recourse to the fluorescent labels and gives access to such structural and biochemical information of the RBC as morphology [12,13,14,15] and concentration of its specific molecules [9,16]. This has allowed new methods of optical sectioning to be explored in microscopy. Methods that use interferometry (e.g., digital holography) are also used to extract a cell’s refractive index [11]. Unfortunately, this class of methods is subject to the constraints of speckle noise due to the coherence of the illumination sources used [17]. Such noise affects the quality of the measurements. An alternative is the use of methods based on illumination sources which are partially coherent such as the method of the transport of intensity equation (TIE). By reformulating TIE and taking into account its link with the refractive index, Phillips et al. [18] performed the three-dimensional (3D) reconstruction of refractive index for microspheres and healthy RBCs. Unfortunately, for the specimens analyzed whose sizes were lower than 0.1 μm or higher than 2.8 μm (which is the case for RBCs), the 3D reconstruction failed [18] due to diffraction. Diffraction not only induces a problem of signal detection, but also cancels the paraxial ray assumptions required to perform TIE. 

In this study, we propose an optical method based on the combination of deconvolution, topography and 3D refractive index reconstruction of RBCs which uses the TIE to differentiate malaria-infected RBCs from healthy RBCs. To our knowledge, this is the first time this technique has been used to identify malaria-infected RBCs. Although similar work has been reported by Park et al. [7], their method was based on a combination of tomographic phase microscopy and diffraction phase microscopy, which both use interferometry. The optical components required for these two techniques increase the cost of the system while interferometry gives rise to speckle noise. The approach that we propose is computationally simple and does not require complicated optical system set-ups. It only requires knowledge of the brightfield microscope’s optical properties which are characterized by its point spread function (PSF). Using partially coherent illumination and the microscope’s 3D PSF, our technique enables us to overcome the constraints of speckle noise as well as cancel diffraction effects in order to highlight the sub-structures of malaria-infected RBCs. This results in clear identification of the stages of infection of RBCs. 

## 2. Materials and Methods

### 2.1. Experimental Set-Up

**Figure 1:**
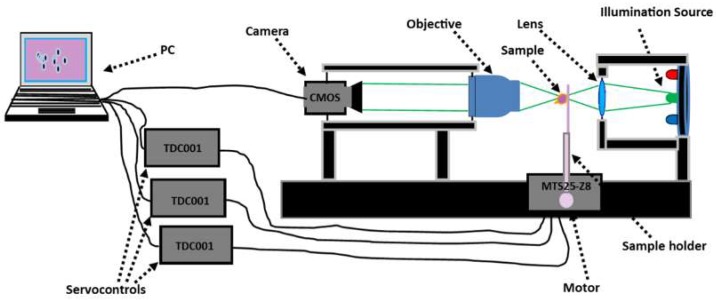
Microscope constructed for brightfield image recording.

Figure 1 shows the experimental set-up. It consisted of a microscope with a dry objective and a 16-bit monochrome complementary metal-oxide semiconductor (CMOS) camera. Its pixel size and resolution were, respectively, 5.5 μm × 5.5 μm and 2048 × 1088 pixels on an effective image chip size of 11.264 mm × 5.984 mm. A set of quasi-monochromatic light emitting diodes (LEDs) was used for illumination. Automated and sequential acquisition of a set of brightfield images was achieved by focusing on the sample along the optical axis using a stepper motor (MTS25-Z8). This allowed the recording of images along several planes of the sample. The portable computer (PC) was connected to three servocontrols (TDC001) which triggered movement of the motor in the *x*, *y* and *z* directions to facilitate image acquisition from any region of interest within the sample. 

### 2.2. Sample Preparation

The samples used in this work were unstained thin blood smears that had been positively identified as infected with malaria. They were cell cultures of *Plasmodium falciparum* parasites provided by the Pastor Institut of Côte d’Ivoire which is a specialized center. The post-infection times for the infected RBCs were: *t* = 8 h for the early ring stage, *t* = 24 h for the trophozoite stage and *t* = 40 h for the schizont stage. Here, the early ring stage was used to appreciate the sensibility of the proposed method. For the different stages of infection of the RBCs, sample preparation was performed and fixed to methanol on different high-purity slides with dimensions 75 mm × 25 mm × 1 mm. Therefore, the sample properties were known a priori. After drying the samples for 25 min, they were used for microscopic examination at the wavelength of λ = 800 nm under partially coherent illumination. The process from measurement to results took 2 min and 30 s. Multiple cells were scanned for each stage of infection. The numbers of scanned cells for the ring stage, trophozoite stage and schizont stage were 68, 42 and 33 respectively.

### 2.3. Measurement Procedure

We recorded the following three sets of images at the *z* plane with illumination wavelength *λ*:

- $TS_{xyz\lambda }$: the image of the sample (sample measurement);
- $TB_{xyz\lambda }$: the image of an empty slide (reference measurement);
- $TD_{xyz\lambda }$: the image of the background which was taken with no slide or sample and with the source illumination turned off.

*x* and *y* are the pixel coordinates. The corrected image $T_{xyz\lambda }$ was obtained from this set of images using flat-field correction [19,20]:

(1) $$T_{xyz\lambda }=\frac{TS_{xyz\lambda }-TD_{xyz\lambda }}{TB_{xyz\lambda }-TD_{xyz\lambda }}$$

### 2.4. Analysis Methods

In the presence of pure phase objects such as RBCs at the wavelength λ = 800 nm, the brightfield image essentially reduces to Equation (2), making it possible to perform linear deconvolution [21,22]:

(2) i=o⊗PSF+N

In Equation (2), i(x, y, z) is the 3D image of the object (o(x, y, z)) while N models the noise, ⊗ is convolution operator. In this study, we first used deconvolution to subtract the contribution of out-of-focus planes and distortions due to the image acquisition system set-up from the images. This made it possible to preserve the TIE paraxial ray assumptions where the diffraction would be prominent [22]. In this approach, an appropriate model of a PSF reflecting the properties of the image acquisition system set-up was required for a deconvolution that would remove diffraction effects and any artefacts. We then proceeded to extract the refractive indices and the topography for the samples by using TIE. In this study, the PSF was computed using the model of Gibson and Lanni [23] but modified and adapted for the use of a dry objective [24]. This computation used both directly and indirectly accessible parameters. The following parameters were directly accessible: numerical aperture of the objective $NA_{ob}=0.75$; working distance of the objective WD=0.71 mm; refractive index of the specimen layer $n_{m}=1.33$; design refractive index of the immersion medium (air) $n_{c^{*}}=1$ and the pixel size in the object space was 0.1 µm. Indirectly accessible parameters were refractive index $n_{c}$ of air at the time of the measurement and the depth $z_{p}$ of the point object in the specimen layer. These were computed by following the approach of Agnero et al. [22]. *n_c_* was determined using Equation (3) [25,26]:

(3) $$n_{c}=1+\left(6.4328\cdot 10^{-5}+\frac{2.94981\cdot 10^{-2}}{146-\lambda^{-2}}+\frac{2.554\cdot 10^{-4}}{41-\lambda^{-2}}\right)\frac{15\cdot P}{T}$$

where *T* and *P* represent, respectively, the temperature in degrees Celsius and pressure in bar at the time of image acquisition, λ is in μm. The measured values of temperature and pressure were, respectively, P=995 hPa and T=18 °C. Using these values in Equation (3) gives $n_{c}=1.00023$. Since the sample was dry, the value of $z_{p}$ was set to 0.

The model of Gibson and Lanni [23] is an accurate model for PSF generation that is suitable for brightfield imaging [22]. It was applied for restoration of the sample images using the Richardson–Lucy algorithm [27,28] with the number of iterations set to 30. The TIE method was implemented as follows:

- The phase $\phi_{z}\left(x,y\right)$ of the wave field traversing the pure phase sample at the wavelength λ wthin a z plane normal to the optical axis was computed using Equation (4) [29,30,31]:
  (4) $$\phi_{z}\left(x,y\right)=TF^{-1}\left[\frac{TF\left[-\;\frac{2\pi }{\lambda }\;\frac{1}{I_{z}\left(x,y\right)}\;\frac{\partial I_{z}\left(x,y\right)}{\partial z}\right]}{4\pi^{2}\left(u^{2}+\nu^{2}\right)}\right]$$
  TF and $TF^{-1}$ are the Fourier Transform and the inverse Fourier Transform respectively. The parameters (u,v) are the spatial frequency variables corresponding to the coordinates (x,y) while $I_{z}\left(x,y\right)$ is the intensity of the wave field within a *z* plane normal to the optical axis.
- The refractive index of each point within the sample is defined by Equation (5) [18]:
  (5) $$\begin{array}{l} n_{z}\left(x,y\right)\\ =n_{m}-TF^{-1}\left\{\frac{u}{u^{2}+\nu^{2}}TF\left\{\frac{1}{{I_{z}}^{2}\left(x,y\right)}\;\frac{\partial I_{z}\left(x,y\right)}{\partial z}\;TF^{-1}\left(\frac{u}{u^{2}+\nu^{2}}\;TF\;\left[\frac{\partial I_{z}\left(x,y\right)}{\partial z}\right]\right)\right\}\right\}\\ +TF^{-1}\left\{\frac{u}{u^{2}+\nu^{2}}TF\left\{\frac{1}{I_{z}\left(x,y\right)}\;TF^{-1}\left(\frac{u}{u^{2}+\nu^{2}}\;TF\;[\frac{\partial^{2}I_{z}\left(x,y\right)}{\partial z^{2}}]\right)\right\}\right\}\\ -TF^{-1}\left\{\frac{\nu }{u^{2}+\nu^{2}}TF\left\{\frac{1}{{I_{z}}^{2}\left(x,y\right)}\;\frac{\partial I_{z}\left(x,y\right)}{\partial z}\;TF^{-1}\left(\frac{\nu }{u^{2}+\nu^{2}}\;TF\left[\;\frac{\partial I_{z}\left(x,y\right)}{\partial z}\right]\right)\right\}\right\}\\ +TF^{-1}\left\{\frac{\nu }{u^{2}+\nu^{2}}TF\left\{\frac{1}{I_{z}\left(x,y\right)}\;TF^{-1}\left(\frac{\nu }{u^{2}+\nu^{2}}\;TF\;\left[\frac{\partial^{2}I_{z}\left(x,y\right)}{\partial z^{2}}\right]\right)\right\}\right\} \end{array}$$
  where $n_{m}$ is the refractive index of the specimen layer. To retrieve the topography of the sample, we used Equation (6) [32]:
  (6) $$h\left(x,y\right)=\frac{\lambda \;\phi \left(x,y\right)}{2\pi \;\left(n_{f}-n_{m}\right)}$$
  ϕ(x,y) is the phase which corresponds to the focal plane and $n_{f}$ is the mean value of the refractive index within the sample in the focal plane.
- We performed measurements at the wavelength λ = 800 nm, because at this wavelength the RBC is a pure phase object [33], making it possible to perform linear deconvolution according to Equation (2). The RBCs essentially consist of hemoglobin (32%), the surrounding membrane (3%) and water (65%) [34]. As such, they can be considered as aqueous solutions whose main solute is the hemoglobin. Therefore, the refractive index of the cell is essentially due to the hemoglobin concentration within the RBC [18,35]. Friebel and Meinke [36] and Tycko et al. [35] showed that a variation in hemoglobin concentration leads to a variation in the refractive index of the cell. These two parameters can be used to confirm whether or not RBCs are healthy or parasitized. The hemoglobin concentration within the RBC is deduced from its refractive index distribution $n_{z}\left(x,y\right)$ using [36]:
  (7) $$C_{Hb}\left(x,y,z\right)=\left(\frac{n_{z}\left(x,y\right)}{n_{water}}-1\right)\frac{1}{\beta \left(\lambda \right)}$$
  where $C_{Hb}\left(x,y,z\right)\;\left(g/\mathrm{dL}\right)$ is the RBC’s hemoglobin concentration, β(λ)=0.001939 dL/g at wavelength λ = 800 nm and $n_{water}$ is the refractive index of water.

## 3. Results and Discussion

### 3.1. Application to Healthy Red Blood Cells

**Figure 2:**
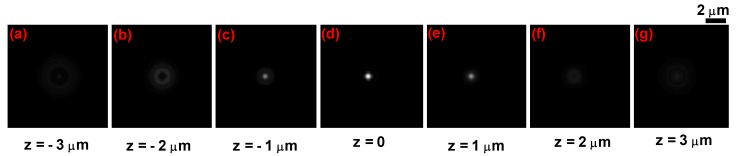
*x*–*y* cross section of the computed three-dimensional (3D) point spread function (PSF) for the brightfield microscope used for the experiment.

Figure 2 shows the computed PSF of the brightfield microscope used in this work for various planes perpendicular to the *z* axis.

**Figure 3:**
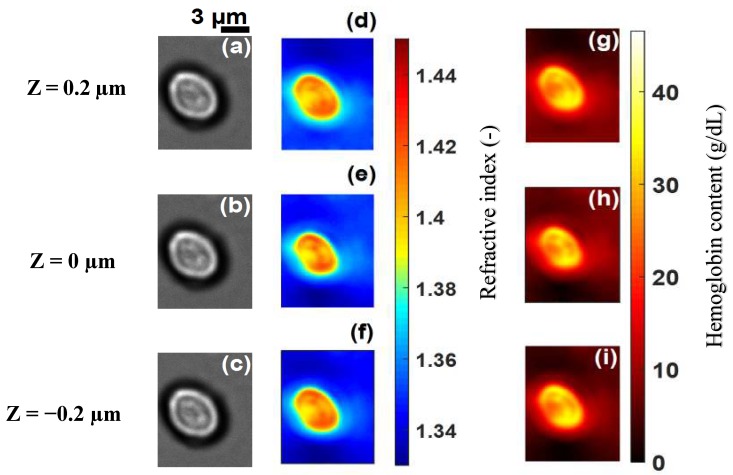
Recorded images of healthy cell **A** at the wavelength λ=800 nm for different *z* planes (**a**–**c**) its corresponding refractive index distribution (**d**–**f**) and hemoglobin content (**g**–**i**). The selected images are representative of a total of 102 healthy cells analyzed.

**Figure 4:**
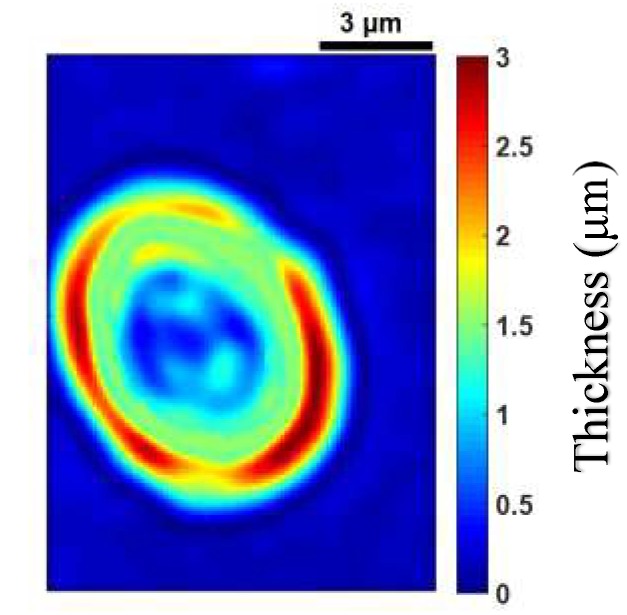
Topography of cell **A**.

**Figure 5:**
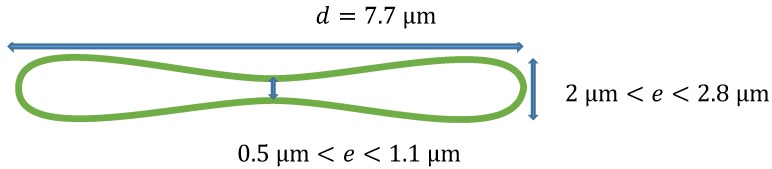
Experimental dimensions for cell **A.**

Figure 3a–c shows images of a healthy RBC (referred to as cell **A**) recorded at a wavelength of 800 nm for three different planes (at z=0.2 µm, z=0 µm and z=−0.2 µm) on the *z* axis. Application of 3D refractive index reconstruction on these images resulted in the refractive index distributions shown in Figure 3d–f for the three planes. Corresponding hemoglobin concentrations are in Figure 3g–i. The refractive index distribution was quasi-homogeneous with a mean value of *n* = 1.408 ± 0.006. The homogeneity of the 3D refractive index distribution within the cell is consistent with what is expected of a healthy RBC [7]. The mean value of the refractive index (1.408 at the wavelength 800 nm) was within the range of refractive index values for healthy RBCs (1.402; 1.409) as reported by Khairullina [37] under the physiological conditions of the cells and by Friebel [33] under conditions close to physiological conditions. The value of 1.408 showed that the RBC **A** (Figure 3a–c) was effectively a healthy cell. The RBC is responsible for the transfer of oxygen and carbon dioxide within the human body. This function is well implemented if the RBC is healthy. This state results in a homogeneous distribution of hemoglobin concentration within the cell. The hemoglobin concentration (Figure 3g–i) extracted from cell **A** was quasi-homogeneous with a mean value of $C_{Hb}=30.25\pm 2.8\;g/\mathrm{dL}$. This value was in agreement with the value $C_{Hb}=30.9\;g/\mathrm{dL}$ reported in the literature [7]. It provided further confirmation that cell **A** was a healthy RBC. Indeed, the hemoglobin concentration of the cell was within the range of values (28 g/dL; 36 g/dL) corresponding to a healthy RBC [7,36]. The mean value of refractive index $n_{f}=1.408$ for the RBC was used to retrieve its topography (Figure 4) using Equation (6). This confirmed the biconcave shape of the healthy cell [7,38]. The edges of the cell were thicker than the center. The thickness of the edges was between 2 µm and 2.8 µm and that of the center varied between 0.5 µm and 1.1 µm. These reported dimensions in Figure 5 were compatible with the dimensions of a healthy RBC in the literature [7,38].

### 3.2. Application to Malaria-Infected Red Blood Cells

**Figure 6:**
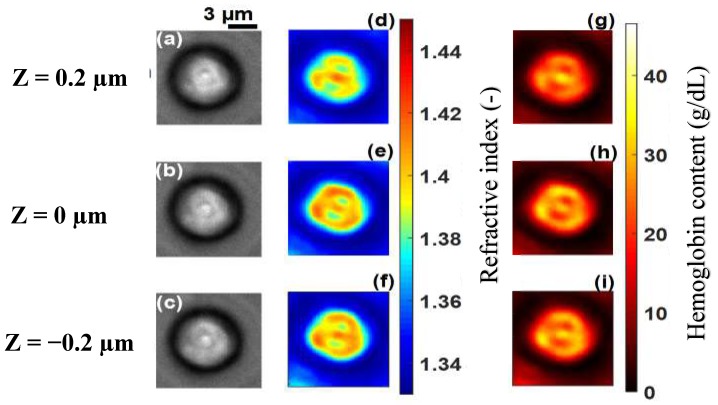
Recorded images of infected cell **B** at an early stage, at the wavelength λ = 800 nm for different *z* planes (**a**–**c**), its corresponding refractive index distribution (**d**–**f**) and hemoglobin content (**g**–**i**). The selected images are representative of a total of 68 cells at an early stage.

**Figure 7:**
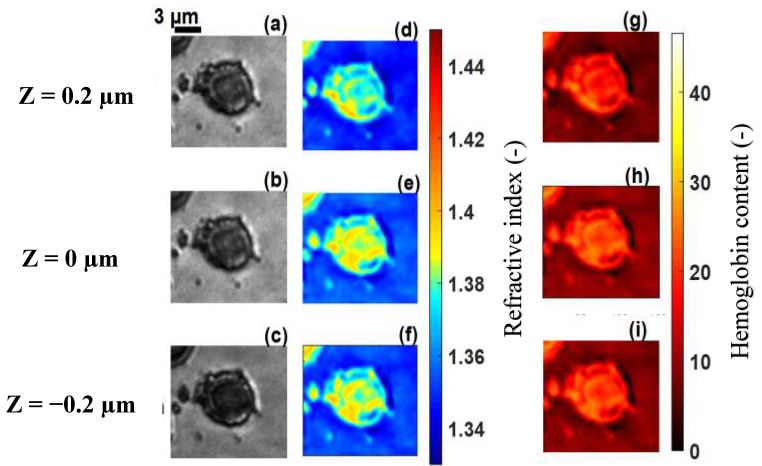
Recorded images of infected cell **C** at the trophozoite stage, at the wavelength λ=800 nm for different z planes (**a**–**c**), its corresponding refractive index distribution (**d**–**f**) and hemoglobin content (**g**–**i**). The selected images are representative of a total of 42 cells analyzed.

**Figure 8:**
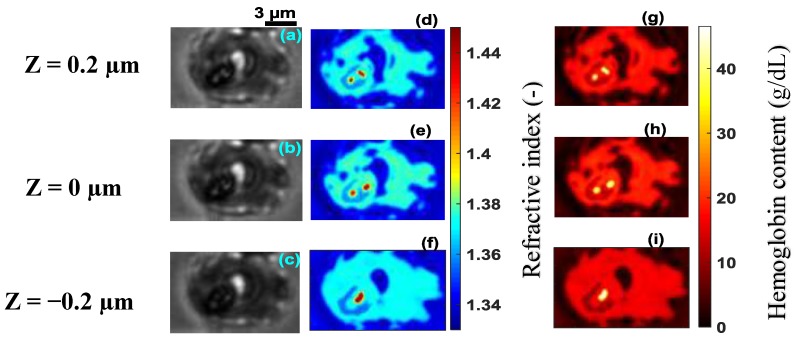
Recorded images of infected cell **D** at the schizont stage, at the wavelength λ=800 nm for different z planes (**a**–**c**), its corresponding refractive index distribution (**d**–**f**) and hemoglobin content (**g**–**i**). The selected images are representative of a total of 33 cells analyzed.

**Figure 9:**
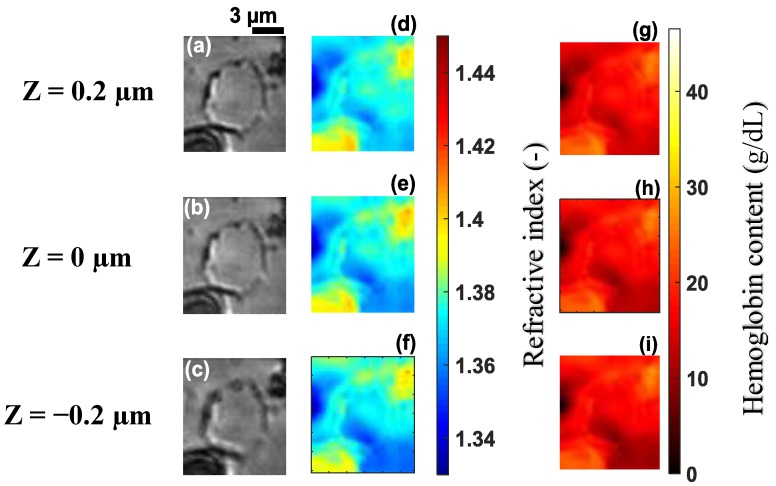
Recorded images of infected cell **E** at the schizont stage, at the wavelength λ=800 nm for different z planes (**a**–**c**), its corresponding refractive index distribution (**d**–**f**) and hemoglobin content (**g**–**i**). The selected images are representative of a total of 28 cells analyzed.

The technique was applied to malaria-infected RBCs, referred to as cell **B**, cell **C**, cell **D** and cell **E** at different stages of infection (Figure 6a–c, Figure 7a–c, Figure 8a–c and Figure 9a–c). Figure 6d–f, Figure 7d–f, Figure 8d–f and Figure 9d–f, show the refractive index distributions within the cells. Figure 6g–i, Figure 7g–i, Figure 8g–i and Figure 9g–i present the hemoglobin content of cells **B**, **C**, **D** and **E** respectively. When the RBCs are healthy, hemoglobin is the major component within the cells [34]. The healthy cell can be physically identified by its biconcave shape, its quasi-uniform hemoglobin concentration and its quasi-homogeneous refractive index distributions with mean values within a well-known range.

The hemoglobin content and refractive index for cells **B**–**E** showed a heterogeneous spatial distribution within the different cells (Figure 6, Figure 7, Figure 8 and Figure 9). The variation of these parameters is an indicator of the presence of parasites. The asymmetry of the spatial distribution of hemoglobin and refractive index within cells **B**–**E** revealed a significant difference between the healthy RBC and the parasitized RBCs. It is clear that cells **B**–**E** were parasitized. Indeed, the characteristics of the host erythrocyte change as soon as the cell is infected with *Plasmodium falciparum* [39]. This parasite appears in the body through the bite of the female Anopheles mosquito and moves from the liver to the RBCs (erythrocytes). When entering an erythrocyte, the parasite induces structural and biochemical changes in the host cell. The modifications allow the parasite to be fed [40]. During the intra-erythrocytic cycle, hemoglobin, the major component of the host cell, is degraded to serve as nourishment for the parasite, which digests it as a free ferrous heme [41,42]. This is rapidly transformed into ferric heme, which is highly toxic to the parasite. Consequently, the cells lose their homogeneous structure due to the presence of parasites. This explains the asymmetry of the hemoglobin content and the refractive index distribution within the different parasitized cells (Figure 6, Figure 7, Figure 8 and Figure 9). The parasite triggers a detoxification process of the ferric heme by biocrystallizing it into a complex called hemozoin, which is the malaria pigment. The red areas with a refractive index greater than 1.42 in Figure 8d–f indicate the presence of hemozoin. Refractive index values greater than 1.42 have been reported as an indicator of the presence of hemozoin in malaria-infected RBCs in studies by Kyoohyun et al. [8] and Park et al. [7].

**Figure 10:**
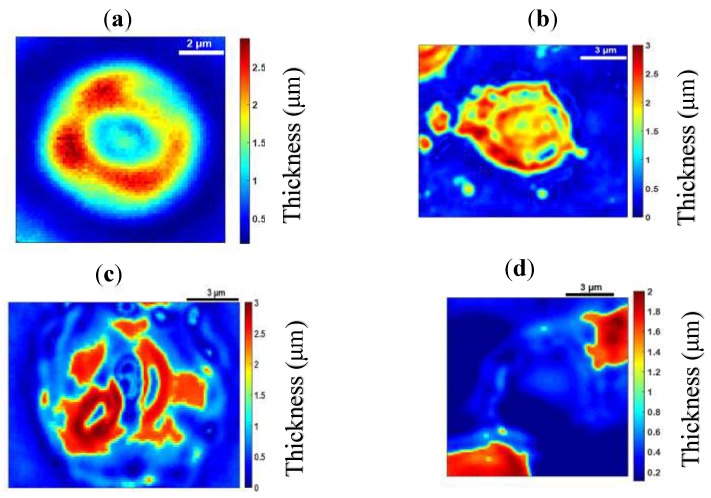
Topography of cell **B** (**a**), cell **C** (**b**), cell **D** (**c**) and cell **E** (**d**).

A parasitized RBC is characterized not only by the optical properties of hemoglobin but also by those of hemozoin and the parasite. The parasite was characterized by regions of low refractive index values within the different cells. In Figure 6, Figure 7, Figure 8 and Figure 9, we can see a decrease of the hemoglobin content and the refractive index values within the parasitized cells. We evaluated the mean values of the refractive indices of the infected cells as well as their hemoglobin content. These are presented in Table 1. The mean values of the refractive indices were used to evaluate the topography (Figure 10) for the parasitized cells via Equation (6). It is clear from Table 1 that refractive index values and hemoglobin contents significantly differ from one infected RBC to another. However, they are quasi-identical for cells **D** and **E**. This assumed that these two cells were at the same stage of infection. When a merozoite enters the erythrocyte, the parasite is surrounded by the membrane in which it grows. It first takes the form of a ring. At this stage, the RBC preserves its biconcave shape [7,43,44,45]. This allowed us to affirm that the infection of cell **B** (Figure 6) was at the ring stage, because the biconcave shape of the host cell was by and large preserved (Figure 10a). The edges of the cell were thicker than the center. The thickness of the edges varied between 1.8 µm and 2.8 µm and the thickness of the center varied between 0.6 µm and 1.2 µm.

The value of the refractive index (1.396) and hemoglobin content (25.59 g/dL) extracted from cell **B** serve to confirm that it is at the ring stage of infection. These extracted values were in agreement with the refractive index value *n* = 1.395 and the range (24 g/dL; 29 g/dL) of hemoglobin content values reported in the literature [7].

**Figure 11:**
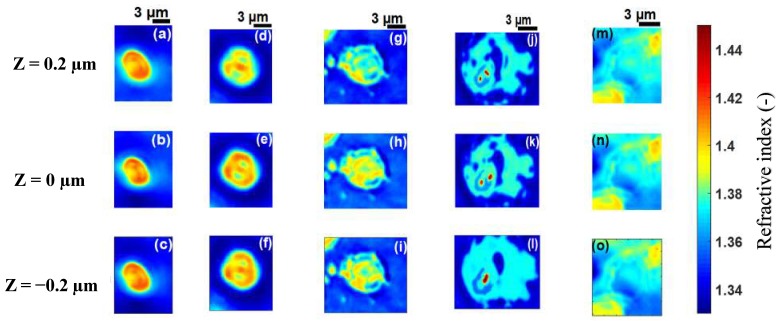
Refractive index distributions within cell **A** (**a**–**c**), cell **B** (**d**–**f**), cell **C** (**g**–**i**), cell **D** (**j**–**l**), and cell **E** (**m**–**o**) for different z planes.

From the ring stage, the parasite matures to the trophozoite stage where the metabolism becomes more intense [46]. At this stage, the RBC loses its biconcave shape [43,47] as can be seen in Figure 10b–d which are topographies of cells **C**, **D** and **E**, respectively. Each of these cells had a topography that was significantly different from healthy cell **A** (Figure 4a) and cell **B** (Figure 10a) which both had a biconcave shape. The loss of biconcavity of host cells **C**, **D** and **E** can be clearly seen in Figure 10. This modification was due to the invasion of the parasites whose metabolic activity within these cells was prominent at these stages of infection, resulting in their deformation [48]. The mean value of refractive index 1.381 for cell **C** (Figure 7) is close to the mean value of 1.383 for an infected RBC at the trophozoite stage which was reported by Park et al. [7]. Finally, at 40 h post-invasion, the parasite reaches the schizont stage, corresponding to a rapid phase resulting in the formation of 8–32 new merozoites. At this stage, the infected RBC explodes, releasing merozoites ready to infect new RBCs. This stage of infection corresponds to cell **D** (Figure 8a–c), which is clearly in decomposition. The refractive index (1.371) and the hemoglobin concentration (15.90 g/dL) of cell **D** confirmed this schizont stage infection. These values were in agreement with the values *n* = 1.373 and $C_{Hb}$ = 18.7 g/dL reported in the literature [7,49]. At the schizont stage, the 3D refractive index distribution within the cell showed some areas characterized by high refractive index values (Figure 8d–f), indicating the presence of hemozoin. This results from the intense metabolic activity of the parasite at an advanced stage of its growth. As it grows within the host cell, the parasite transforms hemoglobin into hemozoin which is identified by a refractive index distribution greater than *n* = 1.42 [8]. At this stage of infection, the hemoglobin content of the cell undergoes a significant decrease. It is known that the parasite can consume up to 80% of the hemoglobin content in the host cell [50]. The decrease in hemoglobin content resulted in a 3D distribution of decreasing refractive index values according to Equation (7). This had a major influence on the morphological structure of infected cells at this advanced stage. At this stage, if the patient is untreated, all parasites gradually grow at the same rate. It is then said that they are becoming synchronous. For effective treatment which would automatically reduce the healthcare cost, it is critical to diagnose malaria in the early stage. This requires more sensitive and rapid techniques. However, detecting changes in host RBC before the parasite matures beyond the ring stage—its earliest stage, and the only stage found in circulating blood—is difficult. In this study, the early ring stage was used with the primary aim of appreciating the sensibility of the proposed method. Changes detected inside the cell in the early ring stage (Figure 11d–f) revealed the sensibility of the technique.

**Figure 12:**
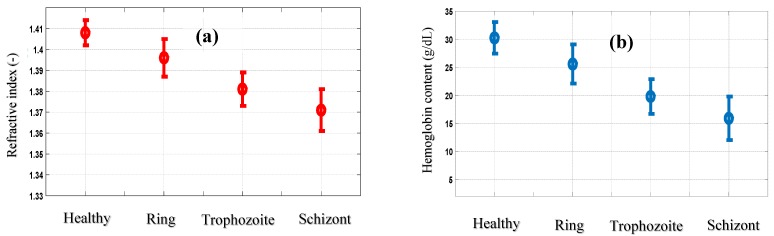
The refractive index of RBCs (**a**) and hemoglobin content (**b**) for the different stages of infection.

Our results showed that the lower the refractive index (Figure 11), the more advanced the infection of the cell (Figure 12a), resulting in a decrease in its hemoglobin content (Figure 12b). The optical and physical characteristics, refractive index of 1.372 and hemoglobin concentration of 16.28 g/dL for cell **E** (Figure 9) revealed that it was in the schizont stage of infection.

## 4. Conclusions

In this study, we developed a technique to differentiate malaria-infected RBCs from healthy RBCs. This technique was based on the combination of deconvolution, topography and 3D refractive index extracted from the cells using the computationally simple TIE method. The technique was firstly applied to a healthy RBC, leading to extraction of its optical, physical and biochemical parameters, refractive index, topography and hemoglobin content. The healthy RBC was characterized by its biconcave shape, its quasi-uniform hemoglobin concentration within a range of values consistent with the reported values in the literature, and its quasi-uniform distribution of refractive indices within a range of values compatible with the reported values in the literature. The technique was also applied to parasitized RBCs, with the results showing a significant difference between the healthy RBC and the parasitized RBCs. The parasitized cells were characterized by the loss of homogeneity of their hemoglobin content and refractive index distribution. The values of these parameters were low due to the presence of the parasites which fed on the hemoglobin. The topography of the cells and their optical and biochemical parameters made it possible to identify life stages of the parasite. This work introduces a significant method of analyzing malaria-infected RBCs at a lower cost and without the use of fluorescent labels for the parasites.

## Figures and Tables

**Table 1:** Mean values of the refractive indices and the hemoglobin concentration within infected red blood cells (RBCs).

	Cell B	Cell C	Cell D	Cell E
Refractive index mean values (-)	1.396 ± 0.009	1.381 ± 0.008	1.371 ± 0.010	1.372 ± 0.008
hemoglobin content (g/dL)	25.59 ± 3.49	19.78 ± 3.10	15.90 ± 3.88	16.28 ± 3.10
